# Bisphosphonate Treatment of Benign Multifocal and Unifocal Osteolytic Tumours of Bone

**DOI:** 10.1080/1357714031000114165

**Published:** 2003-03

**Authors:** C. L. M. H. Gibbons, M. Petra, R. Smith, N. A. Athanasou

**Affiliations:** Departments of Orthopaedic Surgery and Pathology Nuffield Department of Orthopaedic Surgery University of Oxford Nuffield Orthopaedic Centre Oxford OX3 7LD UK

## Abstract

Growth of benign tumours and tumour-like lesions of bone results in osteolysis which may cause pathological fracture. Bisphosphonates are anti-osteolytic agents which have proved effective in the treatment of number of osteolytic conditions. In this study we report the results of treatment with the aminobisphosphonate, pamidronate, of three benign osteolytic tumours of bone, two cases of fibrous dysplasia and one of Langerhans cell histiocytosis. In all three cases there was clinical and radiological improvement following treatment. Radiologically, bone lesions did not exhibit progressive enlargement. Two cases of fibrous dysplasia also showed features suggestive of increased bone formation. These findings indicate that bisphosphonates are likely to be useful in controlling the osteolysis of benign tumours/tumour-like lesions of bone, particularly in those cases where surgical intervention is not possible or multifocal lesions are present.


*Sarcoma* (2003) 7, 35–41

Taylor & Francis healthsciences

**CASE REPORT**

### Bisphosphonate treatment of benign multifocal and unifocal osteolytic tumours of bone

C. L. M. H. GIBBONS, M. PETRA, R. SMITH & N. A. ATHANASOU

*Departments of Orthopaedic Surgery and Pathology, Nuffield Department of Orthopaedic Surgery, University of Oxford, Nuffield Orthopaedic Centre, Oxford, UK*

**Abstract**
Growth of benign tumours and tumour-like lesions of bone results in osteolysis which may cause pathological fracture. Bisphosphonates are anti-osteolytic agents which have proved effective in the treatment of number of osteolytic conditions. In this study we report the results of treatment with the aminobisphosphonate, pamidronate, of three benign osteolytic tumours of bone, two cases of fibrous dysplasia and one of Langerhans cell histiocytosis. In all three cases there was clinical and radiological improvement following treatment. Radiologically, bone lesions did not exhibit progressive enlargement. Two cases of fibrous dysplasia also showed features suggestive of increased bone formation. These findings indicate that bisphosphonates are likely to be useful in controlling the osteolysis of benign tumours/tumour-like lesions of bone, particularly in those cases where surgical intervention is not possible or multifocal lesions are present.

**Key Words:** *bone, tumour, bisphosphonate, resorption*

### Introduction

The growth of benign tumours and tumour-like lesions of bone is associated with osteolysis. This occurs at a variable rate and may result in bone weakness and pathological fracture. Actively growing benign tumours which are confined within the bone cause expansion and deformity (Stage II lesions) and some (Stage III lesions) are locally aggressive and cause considerable bone destruction.[1] Treatment of enlarging bone tumours is essentially surgical (i.e., excision of the lesion). Complete excision is, however, not always possible and as a consequence the lesion may recur, resulting in progressive bone destruction.

A number of recent studies have reported the use of anti-osteolytic agents such as bisphosphonates to inhibit the progressive osteolysis associated with the growth of benign bone tumours.[2,3] In this paper, we report our experience in using the aminobisphosphonate, pamidronate, to treat three large osteolytic benign tumours (two cases of fibrous dysplasia and one of Langerhans cell histiocytosis) in which surgery was not indicated. Two of these cases had extensive multifocal disease. The results of this treatment were assessed in terms of clinical and radiological evidence of progressive bone destruction and compared with those of previous studies which have used bisphosphonates to treat these lesions.

### Case reports

### *Case 1 – Monostotic fibrous dysplasia*

RC, a 29-year-old housewife presented with debilitating left forearm pain. Fibrous dysplasia was diagnosed at the age of 17 years after multiple pathological fractures involving her left proximal radius. Curettage and grafting of the lesion at this time provided tissue for histological confirmation of the diagnosis of fibrous dysplasia (Fig. 1a). The autologous bone graft was subsequently resorbed (Fig. 1b). She remained symptom-free for 9 years but then developed increasing pain of the left proximal radius over a 12-month period. A bone scan showed modest radionucleotide uptake in a solitary tumour of the radius. Examination of radiographs showed an increase in size of the lesion over 12 years. MRI identified an expansile lytic lesion of the left proximal radius with soft tissue involvement. Features were consistent with the diagnosis of fibrous dysplasia.

Correspondence to: N. A. Athanasou, Departments of Orthopaedic Surgery and Pathology, Nuffield Department of Orthopaedic Surgery, University of Oxford, Nuffield Orthopaedic Centre, Oxford OX3 7LD, UK.

ISSN 1357-714X print/ISSN 1369–1643 online/03/010035-7 © 2003 Taylor & Francis Ltd
DOI: 10.1080/1357714031000114165

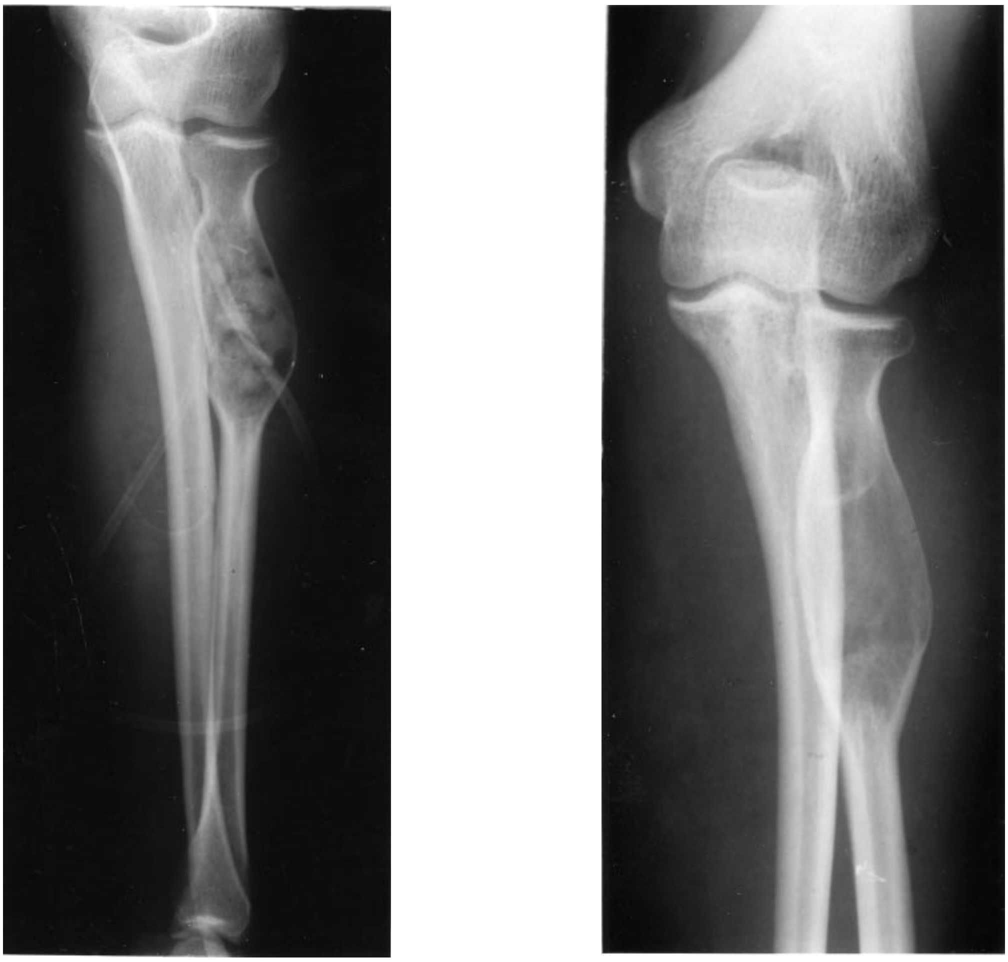

**Fig. 1.** *(a) AP radiograph of the solitary lytic lesion of fibrous dysplasia in the left proximal radius after bone grafting.* (*b*) *AP radiograph of the lesion showing resorption of the bone graft following surgery.*

She underwent three cycles of i.v. pamidronate (30 mg/day for 3 days) at 4-month intervals. She had mild intolerance to pamidronate (chest tightness) after the first dose but this was treated symptomatically and she suffered no further side effects. The bisphosphonate therapy made a significant difference to her pain; the pain score, which was 6/10 before treatment, fell to 0/10 after the first cycle and she remained asymptomatic for 2 months after the completion of the third cycle. The serum alkaline phosphatase and urinary hydroxyproline were normal throughout treatment. Compared with pre-treatment radiographs, radiographic evidence of infilling of the bone lesion was noted 6 months after commencing pamidronate therapy (Fig. 2).

*Case 2 – Polyostotic fibrous dysplasia*

KM, a 37-year-old female housekeeper, presented at the age of 33 years with a 2-year history of right groin and knee pain. Prior to her orthopaedic assessment, she had a full gynaecological and general surgical assessment for her groin pain including surgical exploration to exclude a femoral hernia and pelvic/soft tissue malignancy. Past medical history included fracture of the right tibia at the age of 10 years and re-fracture of the tibia 10 months later. A radiological diagnosis of fibrous dysplasia was made at that time and no further investigations or treatment were undertaken. Skeletal survey confirmed the diagnosis of polyostotic fibrous dysplasia affecting the right hemi-pelvis, right proximal and distal femur, right tibia and talus and first ray of the foot. There was no associated endocrinopathy or musculoskeletal abnormality. Radiographs showed extensive bone lysis of the right proximal femur; there was associated cortical thinning and evidence of an incipient fracture. A trucut bone biopsy of the right proximal femur confirmed the diagnosis of fibrous dysplasia and excluded bone malignancy. MRI of her brain (for persistent headaches) was normal. The patient had intramedullary nailing (AO/ASIF unreamed IMN) of the right femur for her impending fracture.

The patient noticed significant improvement of her clinical symptoms immediately after surgery, but 5 months later became symptomatic again

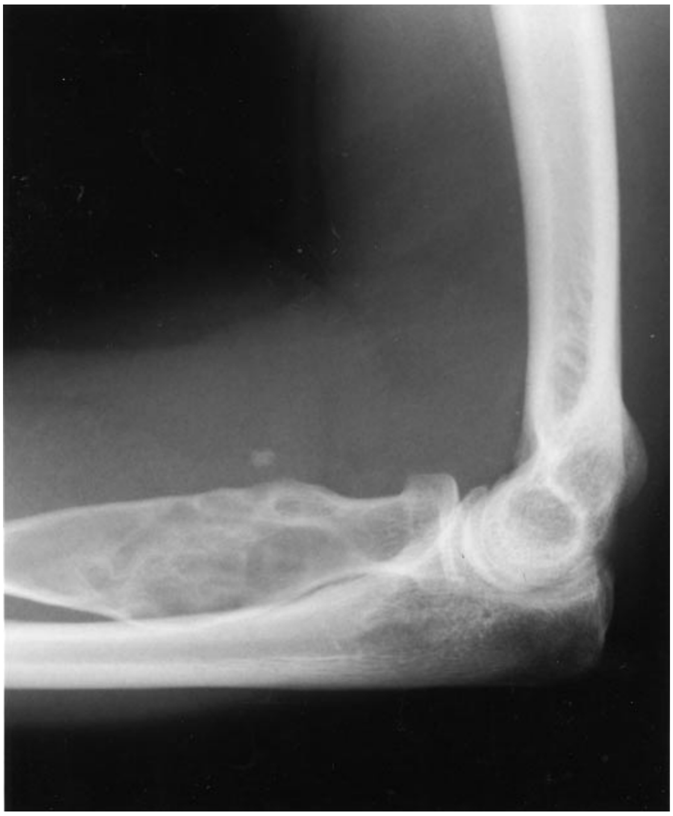

**Fig. 2.** *Radiographic evidence of infilling of the bone lesion following treatment with pamidronate.*

with groin, knee and ankle pain of the right limb at rest and on standing. She was then started on a pamidronate treatment protocol: 30 mg/day for 3 days at 3-month intervals. After the first cycle, she had a florid reaction to pamidronate with increasing pain over a 2-week period. This responded to non-steroidal anti-inflammatory treatment. After the second infusion, she experienced some transient flu-like symptoms. She received a total of eight courses of pamidronate. The pain scores before the initiation of treatment were as follows: right groin 4/10; right hip 6/10; right ankle 6/10. At the latest follow-up, 6 months after the completion of pamidronate, she has no groin or hip pain but continues to have right ankle and mid-foot pain; the latter is most likely secondary to degenerative arthritis of the ankle joint and an associated tibialis posterior tendinopathy. Her pain scores and function have significantly improved with only occasional discomfort, whilst sitting (due to the pelvic lesion). Urine hydroxyproline and serum alkaline phosphatase levels, both of which were elevated, normalised after treatment with pamidronate and have remained normal. In plain radiographs of the femur the lesion has shown progressive remodelling over the 24-month follow-up period with thickening of the surrounding cortex, resolution of the lytic lesion including some infilling of the expanded bone (Figs. 3 and 4).

*Case 3 – Multifocal Langerhans Cell Histiocytosis*

WG, a 33-year-old female clerical worker, presented at the age of 31 years with a 7-month history of left hip pain which was present at night and worse on standing. Clinical examination was unremarkable apart from pain and limited range of movement of the left hip. Past medical history included partial thyroidectomy for thyrotoxicosis and antidepressant treatment.

Radiographs of her pelvis at presentation showed multifocal aggressive lytic lesions of the left acetabulum and the right inferior pubic ramus (Fig. 5). Skeletal survey (including bone scan and MRI) identified a non-painful lytic lesion of the proximal femur and skull. The MRI showed that these lesions were associated with a soft tissue mass. A CT-guided biopsy of the pelvic tumour was inconclusive and an open incisional biopsy of the left pelvic lesion confirmed the diagnosis of Langerhans cell histiocytosis. Following frozen section histological conformation of the diagnosis, she had an intra-operative injection of steroids into the cavity of the left pelvic lesion. She then commenced systemic prednisolone treatment (20–40 mg daily reducing over 6 months). There was modest symptomatic improvement (in pain relief and the ability to weight bear) for the first 3 months, but after 6 months little clinical or radiological improvement was noted. Steroid therapy

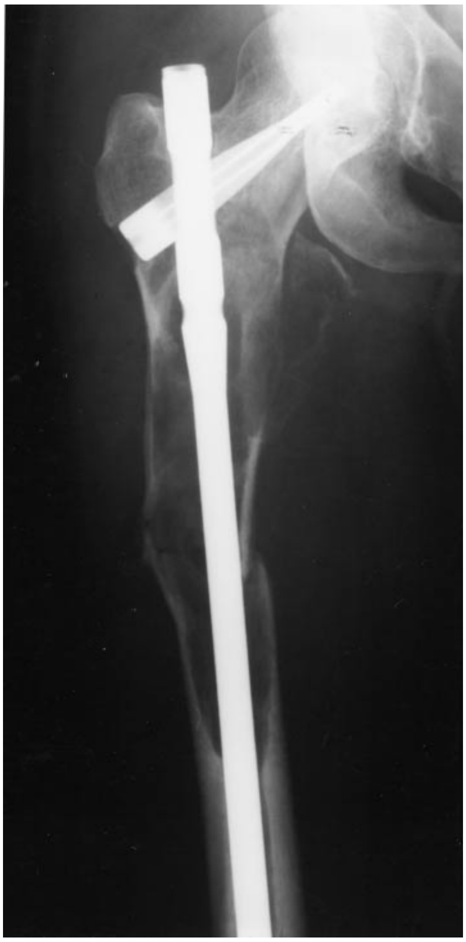

**Fig. 3.** *AP radiograph of the right femur showing a large lesion of fibrous dysplasia with a subtrochanteric pathological fracture. The lesion has been stabilised with an unreamed intramedullary nail.*

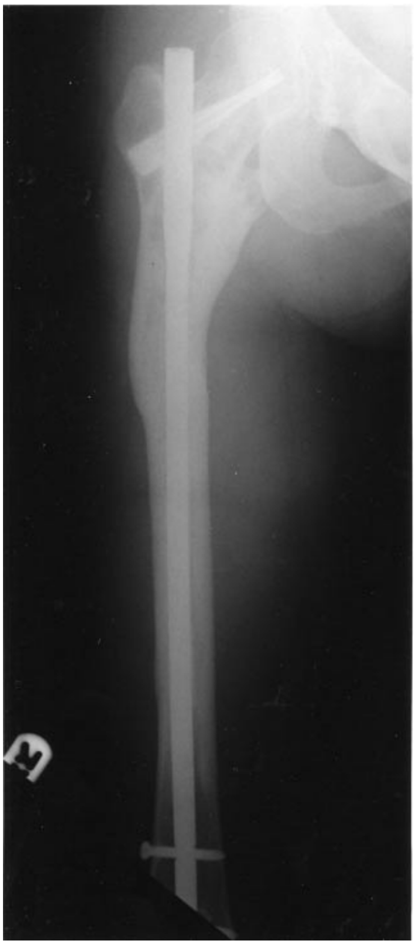

**Fig. 4.** *AP radiograph of the right femur 14 months after surgery and after the patient was commenced on pamidronate therapy.*

was discontinued and a pamidronate protocol, (similar to that used for case 2), was started. The patient had two cycles of i.v. pamidronate (three daily doses of 30, 60 and 60 mg) with intervals of 2 and 5 months. Initially, in the first 6 weeks of pamidronate treatment, she had a dramatic clinical response, the pain score decreasing from 9/10 to 4/10. The pain later returned and modest clinical benefit was noted after the second cycle. Radiologically, although there was little infilling of the large lytic acetabular lesion, the smaller bone lesions showed progressive resolution (Fig. 6). No side effects were noted following pamidronate treatment apart from some initial exacerbation of her bone pain and flu-like symptoms during the second cycle; these were thought to be due to too rapid intravenous administration of pamidronate. Serum alkaline phosphatase remained normal throughout the treatment. Further investigations for diabetes insipidus and pituitary gland function, MRI and CT scan of the pelvis, MRI of the brain were normal. A CT scan of her chest showed nodular pulmonary LCH.

### Discussion

Actively growing benign tumours and tumour-like lesions of bone are associated with increased bone resorption which weakens the integrity of the skeleton. Complete surgical removal or extirpation of these lesions is not always possible and control of the progressive osteolysis and bone weakness that is caused by the growth of these lesions is a significant clinical problem. Bisphosphonate treatment is well-established in controlling bone destruction due to metastatic disease, Paget's disease, osteoporosis and multiple myeloma.[4,5] In this study, we have shown that in three patients, one with monostotic fibrous dysplasia, one with polyostotic fibrous dysplasia and one with multifocal Langerhans cell histiocytosis, treatment with an aminobisphosphonate resulted in inhibition and control of the progressive bone destruction associated with active enlargement of these lesions.

Fibrous dysplasia is a relatively common benign bone lesion which is usually monostotic but in about 20% of cases is polyostotic. Clinically, most patients present with bone pain due to enlargement of the bone lesion. Spontaneous healing of these lesions

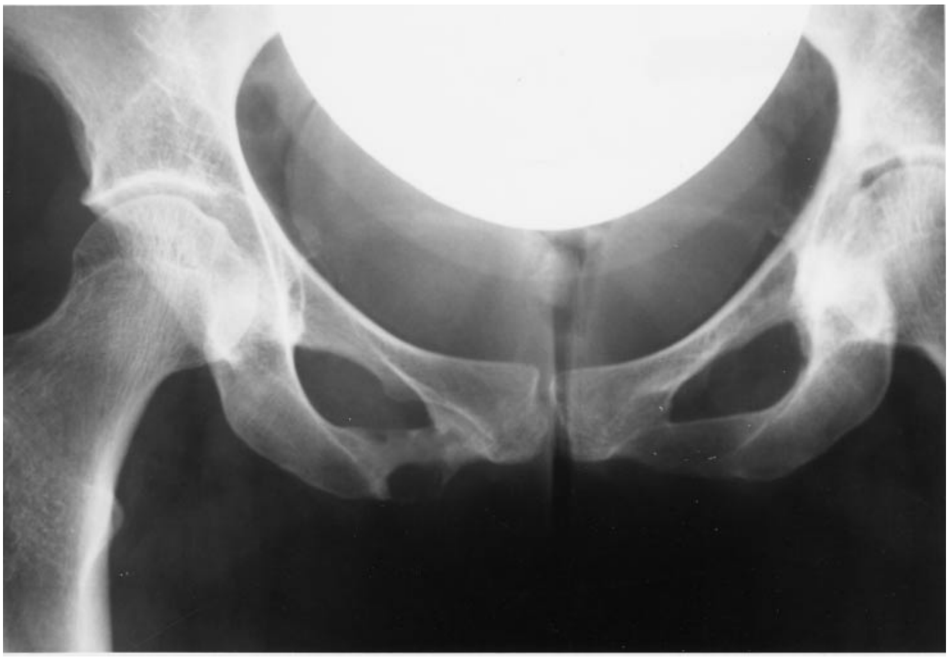

**Fig. 5.** *Multifocal lytic pelvic lesions of Langerhans cell histiocytosis in the left acetabulum and the right inferior pubic ramus.*

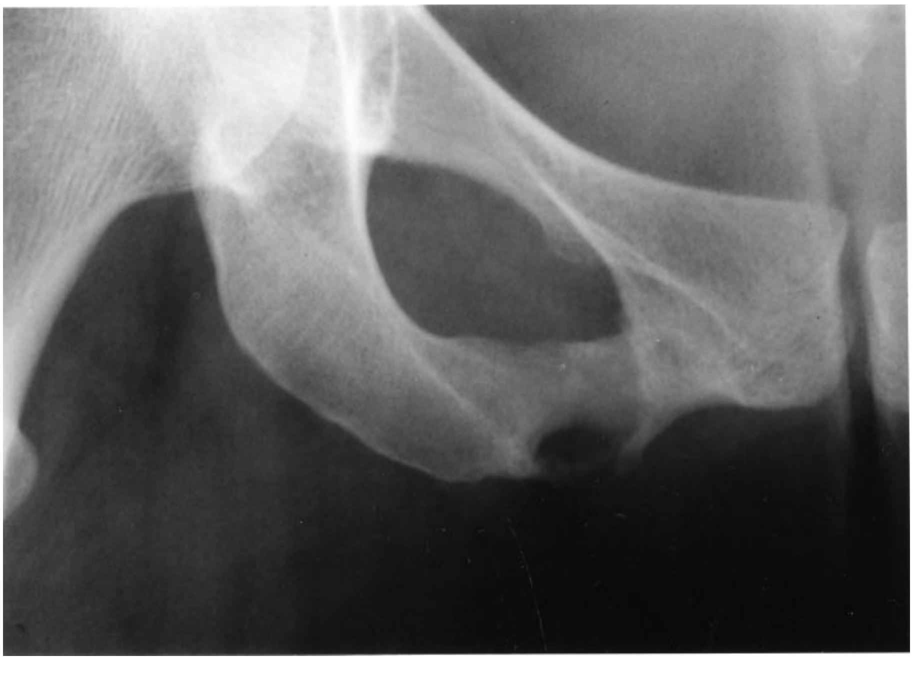

**Fig. 6.** *Ten months after pamidronate treatment radiological evidence of progressive resolution of bone lysis is seen in the lesion in the right inferior pubic ramus.*

does not occur and treatment is generally directed towards pain relief and surgical correction of deformity and pathological fractures.[6,7] Surgical treatment options include various modes of internal fixation and/or bone grafting; this is aimed at providing mechanical support and biomechanical realignment of the affected bones. However, bone graft (as in Case 1 in this study) is characteristically resorbed as the lesion continues to enlarge. The two cases of fibrous dysplasia which received pamidronate treatment in the present study showed clinical and radiological improvement. Both cases showed some resolution of symptoms including an improvement in the pain score. There was also radiological evidence of bone formation and infilling of the lytic lesion. Liens *et al.*[8] also noted a significant decrease in bone pain in nine patients with symptomatic and severe fibrous dysplasia who were treated with pamidronate and followed-up for a period of 4 years. Some radiological resolution of the osteolytic

**Table 1.** *Clinical details of bisphosphonate-treated cases*

| | Sex/age | Duration of treatment with bisphosphonates | Biochemical markers* | Follow-up duration | Clinical result | Radiological result |
|---|---|---|---|---|---|---|
| Case 1: Monostotic fibrous dysplasia | 29-year-old/ Female | 3-cycles at 4-month intervals | Normal throughout treatment | 18 months | Pain score decreased from 6/10 to 0/10; remains asymptomatic | Progressive infilling of radial lytic lesion |
| Case 2: Polyostotic fibrous dysplasia | 37-year-old/ Female | 8-cycles at 3-month intervals | Normal throughout treatment | 24 months | Pain score decreased from 6/10 to 0/10; remains asymptomatic in 2 out of 3 lesions | Progressive infilling and remodelling of femoral lytic lesion |
| Case 3: Multifocal LCH | 33-year-old/ Female | 2-cycles at 2- and 5-month intervals | Normal throughout treatment | 18 months | Pain score decreased from 9/10 to 4/10 | Progressive resolution of smaller lesions; minimal infilling of large acetabular lesion |

*Alkaline phosphatase and urinary hydroxyproline.

lesions was seen in four of the nine patients in this study. A further report by Chapurlat *et al.*[2] on pamidronate treatment of 20 patients with monostotic and polyostotic fibrous dysplasia also noted significant clinical and radiological improvement of the lesions at an average follow-up of 39 months. The severity of bone pain and the number of painful sites was reduced significantly. Some infilling of osteolytic areas was noted on radiographs in nine of the 20 patients following treatment. All biochemical markers of bone remodelling were lowered significantly. More recently, Lane *et al.*[3] reported findings in six cases of fibrous dysplasia treated with either oral alendronate or intravenous pamidronate and oral alendronate. They reported significant clinical improvement, with an average decrease in the pain score of 75% for the patients who received alendronate and pamidronate at 2 years follow-up. There was also an improvement in the bone quality with four of the six patients showing progressive ossification of the lesion and a decrease in the diameter of the lesions. All the patients who received combination intravenous and oral therapy had a significant decrease in collagen breakdown products (urinary *N*-telopeptide) as compared with the patients who only received oral agents.

Clinical and radiological improvement was also noted following pamidronate treatment of a case of multifocal Langerhans cell histiocytosis (LCH) in the present study. Although the clinical improvement was short-lived, no enlargement of the largest bone lesion was noted and several small volume lesions actually showed progressive radiographic resolution. Langerhans cell proliferation in LCH may produce single or multiple lesion in skeletal tissues.[7] Treatment options for specifically painful and/or radiologically aggressive lesions include curettage, intralesional infiltration of corticosteroids, chemotherapy and radiation therapy. There are two previous reports of administration of bisphosphonates to patients with LCH. Elomaa *et al.*[9] treated two adults with multi-focal LCH with oral clodronate for 6 months and noted pain relief with evidence of regression of the bone lesions and decreased bone resorption after approximately 1 month of treatment. More recently, Farran *et al.*[10] reported the results of bisphosphonate treatment in a 14-year-old boy with multifocal LCH resistant to chemotherapy, steroids, non-steroidal anti-inflammatory drugs for NSAID and narcotic analgesics. The patient responded well to two cycles of i.v. pamidronate and remained pain-free 4 months after his last treatment cycle. An MRI confirmed regression of some of the lesions. The LCH patient in our study showed significant pain relief 6 weeks after initiation of treatment with i.v. pamidronate but this improvement was not maintained. Radiographs taken 1 month after the completion of the second cycle showed some evidence of resolution of the large lytic acetabular lesion. (The patient has declined further investigation, limiting our analysis for studying progression of the lesion following treatment).

The rationale for using bisphosphonates to control the osteolysis associated with the growth of benign tumours in bone is that these compounds inhibit osteoclastic resorption and increased bone turnover without affecting osteoblast function.[5,11,12] Bisphosphonates are chemically stable analogues of inorganic pyrophosphate and have a high affinity for bone mineral.[5,13] This tissue-specific targeting of bisphosphonates to bone mineral, especially to sites of osteoclast activity, is extremely useful in controlling the growth of bone lesions. Tumour osteolysis is mediated by osteoclasts and bisphosphonates are found in high concentration beneath osteoclasts at sites of bone resorption (where bone mineral is most exposed) rather than at sites of bone formation.

Bisphosphonates inhibit osteoclast-mediated bone resorption in several ways.[5,13] Binding of bisphosphonates to hydroxyapatite results in changes in the physico-chemical structure of hydroxyapatite crystals. Bisphosphonates also inhibit osteoclast bone-resorbing activity and decrease osteoclast formation from mononuclear phagocyte precursors;[14,15] they also increase apoptosis in osteoclasts with consequent loss of attachment to bone.[16] Bisphosphonates do not inhibit bone formation.[5,11] They do not significantly influence the formation of callus in fracture repair and in general increase the mechanical strength of bone following fracture.[12] As skeletal growth is also not greatly affected, bisphosphonates are thus likely to prove useful agents in the treatment of bone osteolysis associated with bone tumours, many of which arise in the immature skeleton of young patients.

Bisphosphonates are also recognised to have an analgesic effect in bone tumours[5,13] such as LCH where cells within the lesion are known to produce cytokines and prostaglandins which can stimulate progressive osteoclast resorption and pain.[14,15] The analgesic effect of bisphosphonates is explained on the basis that active osteoclasts produce prostaglandins and interleukins that mediate pain (even in the absence of bone damage) and pamidronate, in addition to being a direct osteoclast inhibitor, is known to decrease the production of cytokines including interleukin-1, interleukin-6 and tumour necrosis factor $\alpha$.[5,17]

In summary, bisphosphonates proved effective in controlling tumour osteolysis and in reducing the pain in two cases of fibrous dysplasia and one of multifocal LCH. A similar rationale has been used for employing other anti-osteolytic agents such as calcitonin to control pathological bone resorption associated with the growth of giant cell granulomas of the jaw.[18,19] The role of bisphosphonates in tumour management would appear to merit further investigation.

### Acknowledgements

The authors wish to thank Caroline Costar for typing the manuscript.

### References

1. Enneking WF. A system of staging musculoskeletal neoplasms. *Clin Orthop* 1986; 204: 9–24.
2. Chapurlat RD, Delmas PD, Liens D, *et al.* Long term effects of intravenous pamidronate in fibrous dysplasia of bone. *J Bone Miner Res* 1997; 12: 1746–1752.
3. Lane JM, Safdar NK, O'Connor WJ, *et al.* Bisphosphonate therapy in fibrous dysplasia. *Clin Orthop* 2001; 382: 6–12.
4. Bonjour J-P, Rizzoli R, Ammann P, Chevalley T. Bisphosphonates in clinical medicine. In: Heersche JNM, Kanis J, eds. *Bone Mineral Research 18.* Amsterdam: Elsevier, 1994; 205–263.
5. Rogers MJ, Gordon S, Benford HL, *et al.* Cellular and molecular mechanisms and action of bisphosphonates. *Cancer* 2000; 88: 2961–2978.
6. Harris WH, Dudley HR, Barry RJ. The natural history of fibrous dysplasia. An orthopaedic, pathological and roentgenographic study. *J Bone Joint Surg* 1962; 44A: 207–233.
7. Unni KK. *Dahlin's Bone Tumours. General Aspects and Data on 11,087 Cases.* New York: Lippincott Raven, 1996.
8. Liens D, Delmas PD, Meunier PJ. Long term effects of intravenous pamidronate in fibrous dysplasia of bone. *Lancet* 1994; 343: 953–954.
9. Elomaa I, Blomqvist C, Porkka L. Experiences of clodronate treatment of multifocal eosinophilic granuloma of bone. *J Intern Med* 1989; 1: 59–61.
10. Farran RP, Zaretski E, Egeler M. Treatment with Langerhans cell histiocytosis with pamidronate. *J Pediatr Hematol* 2001; 23: 54–56.
11. Abildgaard N, Rungby J, Glerup H, *et al.* Long term oral pamidronate treatment inhibits osteoclastic bone resorption without affecting osteoblastic function in multiple myeloma. *Eur J Haematol* 1998; 61: 128–134.
12. Fleisch H. Can bisphosphonates be given to patients with fractures? *J Bone Mineral Res* 2001; 16: 437–440.
13. Lin JH. Bisphosphonates: a review of their pharmacokinetic properties. *Bone* 1996; 18: 75–85.
14. Sato M, Glasser W. Effects of bisphosphonates on isolated rat osteoclasts as examined by reflected light microscopy. *J Bone Miner Res* 1990; 5: 31–40.
15. Nishigawa M, Akatsu T, Katayama Y, *et al.* Bisphosphonates act on osteoblast cells and inhibit osteoclast formation in mouse marrow cutures. *Bone* 1996; 18: 9–14.
16. Hughes DE, Wright KR, Uy HL, *et al.* Bisphosphonates promote apoptosis in murine osteoclasts in vitro and in vivo. *J Bone Miner Res* 1995; 10: 1478–1487.
17. Sauty A, Peccharstarfer M, Zimmer-Roth I, *et al.* Interleukin-6 and tumour necrosis factor-$\alpha$ levels after bisphosphonate treatment in vitro and in patients with malignancy. *Bone* 1996; 18: 133–139.
18. Flanagan AM, Tinkler SMB, Horton MA, Williams DM, Chambers TJ. The multinucleated cells in giant cell granulomas of the jaw are osteoclasts. *Cancer* 1988; 62: 1139–1145.
19. Harris M. Central giant cell granulomas of the jaw regress with calcitonin therapy. *Br J Oral Maxillofacial Surg* 1993; 31: 89–94.
